# Value of ^18^F-FDG PET/CT-Based Radiomics Nomogram to Predict Survival Outcomes and Guide Personalized Targeted Therapy in Lung Adenocarcinoma With EGFR Mutations

**DOI:** 10.3389/fonc.2020.567160

**Published:** 2020-11-11

**Authors:** Bin Yang, Hengshan Ji, Jing Zhong, Lu Ma, Jian Zhong, Hao Dong, Changsheng Zhou, Shaofeng Duan, Chaohui Zhu, Jiahe Tian, Longjiang Zhang, Feng Wang, Hong Zhu, Guangming Lu

**Affiliations:** ^1^ Department of Medical Imaging, Affiliated Jinling Hospital, Medical School of Nanjing University, Nanjing, China; ^2^ Department of Nuclear Medicine, Affiliated Jinling Hospital, Medical School of Nanjing University, Nanjing, China; ^3^ College of Medical Imaging, Xuzhou Medical University, Xuzhou, China; ^4^ Institute of Precision Medicine, GE Healthcare China, Shanghai, China; ^5^ Department of Nuclear Medicine, Peking Union Medical College Hospital, Beijing, China; ^6^ Department of Nuclear Medicine, The Chinese People's Liberation Army (PLA) General Hospital, Beijing, China; ^7^ Department of Nuclear Medicine, First People’s Hospital of Nanjing, Nanjing, China

**Keywords:** lung adenocarcinoma, positron emission tomography/computed tomography, radiomics, nomogram, targeted therapy

## Abstract

**Objectives:**

To investigate the development and validation of a radiomics nomogram based on PET/CT for guiding personalized targeted therapy in patients with lung adenocarcinoma mutation(s) in the *EGFR* gene.

**Methods:**

A cohort of 109 (77/32 in training/validation cohort) consecutive lung adenocarcinoma patients with an *EGFR* mutation was enrolled in this study. A total of 1672 radiomic features were extracted from PET and CT images, respectively. The least absolute shrinkage and selection operator (LASSO) Cox regression was used to select the radiomic features and construct the radiomics nomogram for the estimation of overall survival (OS), which was then assessed with respect to calibration and clinical usefulness. Patients with an *EGFR* mutation were divided into high- and low- risk groups according to their nomogram score. The treatment strategy for high- and low-risk groups was analyzed using Kaplan–Meier analysis and a log-rank test.

**Results:**

The C-index of the radiomics nomogram for the prediction of OS in lung adenocarcinoma in patients with an *EGFR* mutation was 0.840 and 0.803 in the training and validation cohorts, respectively. Distant metastasis [(Hazard ratio, HR),1.80], metabolic tumor volume (MTV, HR, 1.62), and rad score (HR, 17.23) were the independent risk factors for patients with an *EGFR* mutation. The calibration curve showed that the predicted survival time was remarkably close to the actual time. Decision curve analysis demonstrated that the radiomics nomogram was clinically useful. Targeted therapy for patients with high-risk *EGFR* mutations attained a greater benefit than other therapies (*p* < 0.0001), whereas the prognoses of the two therapies were similar in the low-risk group (*p* = 0.85).

**Conclusions:**

Development and validation of a radiomics nomogram based on PET/CT radiomic features combined with clinicopathological factors may guide targeted therapy for patients with lung adenocarcinoma with *EGFR* mutations. This is conducive to the advancement of precision medicine.

## Introduction

Lung cancer is the leading cause of cancer deaths in the world and has the highest morbidity and mortality rates among all malignant tumors (1, 2). Non-small cell lung cancer (NSCLC) accounts for 85% of all lung cancers (3, 4). Due to the lack of early clinical symptoms, lymph node metastasis or distant metastasis has already occurred by the time of diagnosis, and it is usually too late for surgical intervention (5, 6). Although the prognosis of lung cancer has improved significantly with improvements in treatment methods, the 5-year survival rate for lung cancer patients remains at 17–18% (7, 8).

The tumor, node, and metastasis (TNM) staging system is currently the most valuable and commonly used tumor staging system for assessing the prognosis of malignant tumors (9–12). However, in clinical practice, it is found that the TNM staging system continues to have many shortcomings in the prognostic evaluation of lung cancer. The survival time of patients at the same stage may differ. Therefore, a TNM-based one-size-fits-all strategy might not be suitable for all patients. In addition, it is not currently possible to fully predict the progression and outcome of disease in patients with NSCLC. Therefore, identification of patients at high risk of death would be valuable for guiding therapy (13–15). New methods of prognostic assessment are urgently needed to achieve personalized treatment. A nomogram is an intuitive chart prepared by establishing a statistical prediction model, which includes important tumor prognosis factors. A nomogram is regarded as a tool for quantifying risks and has become the focus of cancer research (16–18).

The^18^F-fluordeoxyglucose positron emission tomography/computed tomography (^18^F-FDG PET/CT) can provide functional, metabolic, anatomical, and morphological imaging. Its’ metabolic parameters can reflect the metabolism of tumor tissue. Studies have shown that FDG uptake in primary tumors is an independent risk factor for patients with early NSCLC (19, 20), although the value of the prognosis in evaluation of advanced NSCLC patients remains controversial (21, 22). Moreover, the ^18^F-FDG PET/CT features of lung cancer are significantly correlated with T stages, N status, pathological stages, and tumor grades (23–25). Therefore, it has been widely used in the diagnosis, staging, and monitoring of the therapeutic effects and prognostic evaluation of NSCLC (26). Radiomics is the high-throughput extraction and analysis of quantitative features from images. Consequently, the prognostic evaluation of NSCLC by PET/CT can be improved (27). Currently, several attempts have been made to improve the performance of predictive models. However, the prognostic prediction performance of radiomics models in these studies was generally poor. Thus the prognostic performance of radiomics has room for further improvement (15, 28). A few studies have evaluated the use of ^18^F-FDG PET/CT radiomics features to predict the NSCLC prognosis; nevertheless the effect of the driver gene mutation status and treatment methods was ignored. The prognosis of patients with NSCLC is closely related to the driving gene mutation status and treatment. So, it is necessary to conduct independent research with these patients to achieve individualized treatment.

The main purpose of this study was to develop a radiomics nomogram based on ^18^F-FDG PET/CT radiomic features combined with clinicopathological factors to predict the survival outcomes of patients diagnosed with lung adenocarcinoma with an epidermal growth factor receptor (*EGFR*) mutation. We also endeavored to provide guidance for treatment strategies and prognostic evaluation of patients with an *EGFR* mutation.

## Materials and Methods

### Patients

The institutional review board of Affiliated Jinling Hospital, Medical School of Nanjing University approved this retrospective study and waived the requirement to obtain informed consent from the patients. In our retrospective investigation, the following inclusion criteria were applied to select patients from the medical database: a) an ^18^F-FDG PET/CT examination within 1 month prior to surgery or biopsy, b) no anti-tumor treatment received before the ^18^F-FDG PET/CT examination, c) with surgical or biopsy specimens confirmed by pathology, and d) with *EGFR* mutation detection results. The exclusion criteria were as follows: a) patients with partial loss of PET or CT images, b) patients with metastases in the lung, and c) images with unclear boundaries of the tumor that could not be accurately delineated.

Altogether, 174 consecutive lung adenocarcinoma patients were identified by applying the above-mentioned inclusion/exclusion criteria from the institutional database between July 2009 and August 2016, and 109 cases were patients with an EGFR mutation. Among those with EGFR mutations, 44 had the 19DEL, 61 had the 21L858R-mutation and four had other EGFR mutations sites. We randomly divided patients with the EGFR mutation into training (n = 77) and validation (n = 32) cohorts following a 7:3 ratio. The clinicopathological data obtained from medical records included age, sex, family history, smoking history, histological grade, lymph node metastasis, distant metastasis, TNM stage (defined according to the eighth edition of the TNM classification and staging system by the American Joint Committee on Cancer), thyroid transcription factor-1 (TTF-1) (− or one + was defined as negative, ≥two + was defined as positive), Ki-67 (≤25% was defined as low expression and >25% as high expression), carcinoembryonic antigen (CEA), and PET/CT metabolic parameters (**Table 1**). The follow-up time was from July 2009 to January 2019. The endpoint of this study was overall survival (OS), which was defined as the time from the date of the ^18^F-FDG PET/CT examination to the date of telephone follow-up or the date of the patient’s death.

**Table 1:** Characteristics of the training and validation cohorts.

Characteristics	Training cohort (n = 77)	Validation cohort (n = 32)	Total(n = 109)	*p*-value
**Gender-no.(%)**				1.000
**Female**	43 (55.844)	18 (56.250)	61 (55.963)	
**Male**	34 (44.156)	14 (43.750)	48 (44.037)	
**Age, mean(SD)**	60.078 (9.373)	60.625 (8.051)	60.239 (8.972)	0.773
**Family history-no.(%)**				0.036
**No**	75 (97.403)	27 (84.375)	102 (93.578)	
**Yes**	2 (2.597)	5 (15.625)	7 (6.422)	
**Smoking status-no.(%)**				0.835
**Non-smokers**	57 (74.026)	25 (78.125)	82 (75.229)	
**Smokers**	20 (25.974)	7 (21.875)	27 (24.771)	
**Histologic grade-no.(%)**				0.376
**Poorly differentiated**	33 (42.857)	13 (40.625)	46 (42.202)	
**Moderately differentiated**	35 (45.455)	12 (37.500)	47 (43.119)	
**Well differentiated**	9 (11.688)	7 (21.875)	16 (14.679)	
**Lymph node metastasis-no.(%)**				0.157
**Yes**	62 (80.519)	21 (65.625)	83 (76.147)	
**No**	15 (19.481)	11 (34.375)	26 (23.853)	
**Distant metastasis-no.(%)**				0.455
**Yes**	53 (68.831)	25 (78.125)	78 (71.560)	
**No**	24 (31.169)	7 (21.875)	31 (28.440)	
**Stage-no.(%)**				0.988
**I/II**	9 (11.688)	3 (9.375)	12 (11.009)	
**III/IV**	68 (88.312)	29 (90.625)	97 (88.991)	
**TTF-1-no.(%)**				0.610
**Positive**	56 (72.727)	21 (65.625)	77 (70.642)	
**Negative**	21 (27.273)	11 (34.375)	32 (29.358)	
**Ki-67-no.(%)**				1.000
**≤25%**	49 (63.636)	20 (62.500)	69 (63.303)	
**>25%**	28 (36.364)	12 (37.500)	40 (36.697)	
**CEA-no.(%)**				0.344
**≤2.60**	18 (23.377)	11 (34.375)	29 (26.606)	
**>2.60**	59 (76.623)	21 (65.625)	80 (73.394)	
**SUVmax-no.(%)**				0.198
**≤5.33**	20 (25.974)	13 (40.625)	33 (30.275)	
**>5.33**	57 (74.026)	19 (59.375)	76 (69.725)	
**SUVmean-no.(%)**				0.511
**≤1.74**	7 (9.091)	5 (15.625)	12 (11.009)	
**>1.74**	70 (90.909)	27 (84.375)	97 (88.991)	
**TLG(g)**				0.880
**≤54.02**	51 (66.234)	20 (62.500)	71 (65.138)	
**>54.02**	26 (33.766)	12 (37.500)	38 (34.862)	
**MTV(cm^3^)**				0.824
**≤7.32**	35 (45.455)	16 (50.000)	51 (46.789)	
**>7.32**	42 (54.545)	16 (50.000)	58 (53.211)	

CEA, carcinoembryonic antigen; MTV, metabolic tumor volume; SUV_max_, maximal standard uptake value; SUV_mean_, mean standard uptake value; TLG, total lesion glycolysis; TTF-1, thyroid transcription factor-1; EGFR, epidermal growth factor receptor.

### PET/CT Imaging Method, Image Acquisition, and Measurement of Metabolic Parameters

Patients underwent PET/CT imaging (Biography 16, Siemens, Erlangen, Germany) using ^18^F-FDG synthesized by the Canadian EBCO TR19 medical cyclotron and chemical synthesis system. The radiochemical purity was >95%. The patients fasted for 6–8 h before undergoing the scan. Patients were intravenously injected with ^18^F-FDG (3.7–6.66 MBq/kg) and underwent a whole-body PET/CT scan from the skull base to the upper section of the thigh. CT scan parameters were as follows: tube voltage120 kV, Tube current 140 mAs, and layer thickness and layer spacing 5 mm, matrix 512 × 512, and tube rotation speed 0.8 s/r. The PET acquisition parameters were as follows: three-dimensional at 3 min/bed, iterative algorithm, iterations four subsets, eight resolution, 4.1 mm lateral, 4.6 mm axial, matrix 128 × 128, voxel size 5.3 × 5.3 × 5.3 mm^3^. The images were reconstructed using an iterative reconstruction method resulting in CT, PET, and PET/CT fusion images that were transferred to a post-processing workstation. We used Microsoft Viewer software (version VB10, Siemens) to calculate the metabolic parameters on the PET images. PET images were first converted to SUV images in the software without other processing methods. Then, the three-dimensional region of interest (ROI) was manually delineated by a radiologist (YB) to calculate the maximum standard uptake value (SUVmax, with a threshold set to 40%), mean standard uptake value (SUVmean), and metabolic tumor volume (MTV). Subsequently, the total lesion glycolysis (TLG) (TLG = SUVmean × MTV) was calculated.

### EGFR Gene Detection

EGFR genetic mutations were tested from the affected tumor tissue sample obtained by surgical resection or biopsy. The amplification refractory mutation system polymerase chain reaction method was used to detect mutation sites in four exons (exons 18–21) in the coding region of the EGFR gene, the results of which were acquired according to the interpretation principle provided by the reference test kit. If any exon mutation was detected, the tumor was identified as an EGFR mutant; otherwise, the tumor was identified as EGFR wild type.

### Tumor Segmentation

**Figure 1:**
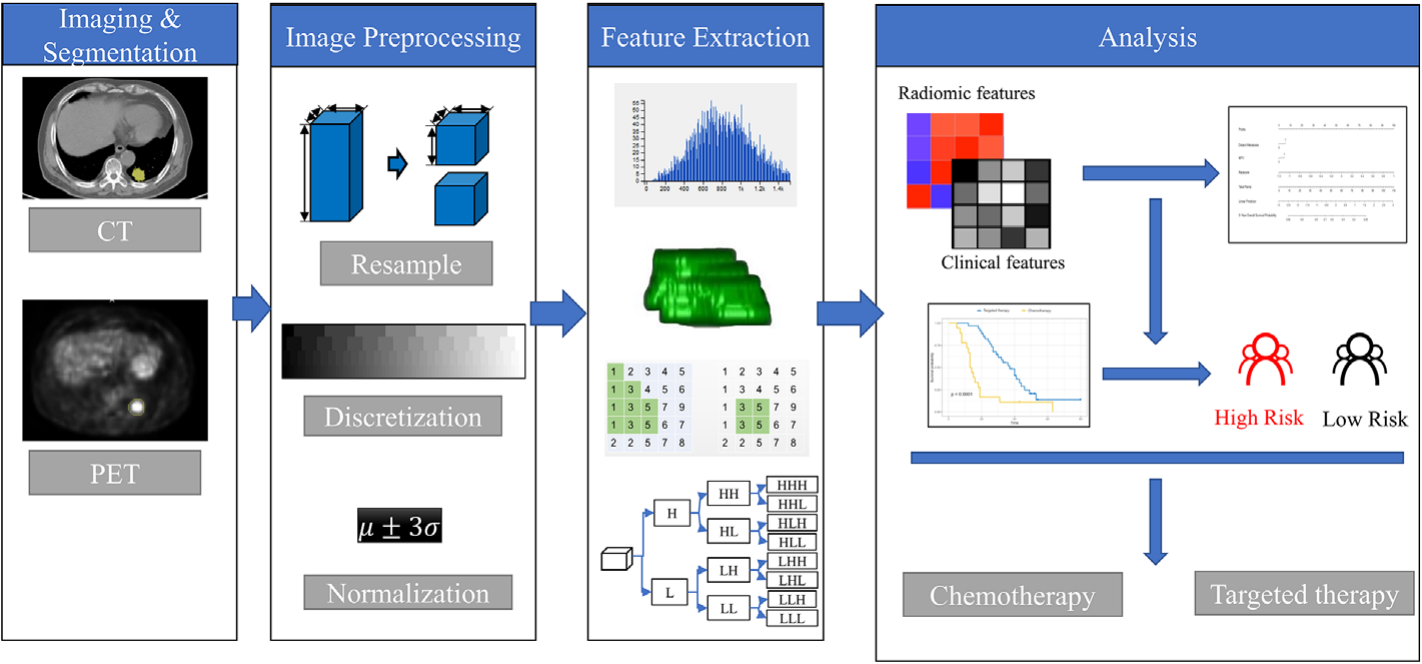
The framework for developing the radiomics nomogram and treatment strategy decisions. The lesions were segmented on Siemens Radiomics prototype semiautomatically, and 1,672 radiomics features, including first order features, shape related features, and texture features were extracted using the software after image pre-processing. The least absolute shrinkage and selection operator (LASSO) Cox regression was used to select radiomics features and clinicopathological factors to construct the radiomics nomogram. Patients with *EGFR* mutations were divided into high- and low-risk groups according to the rad score. The treatment strategy was analyzed in the high- and low-risk groups.

A volume of interest (VOI) segmentation was semiautomatically produced by drawing a line across the boundary of the tumor and manually adjusted by a chest radiologist (YB, 9 years of experience in the lung diagnosis) in a three-dimensional domain using the radiomics prototype (Radiomics, Frontier, Siemens; **Figure 1**) and confirmed by another chest radiologist (JS, 15 years of experience). Then, the tool automatically found the neighboring voxels in 3D space with the same gray level through an automatic algorithm. This is the Random Walker-based lesion segmentation for solid and subsolid lung lesions (29). Both radiologists were blinded to the patients’ clinical information. The details of the tumor segmentation are described in **Appendix 1**.

### Feature Extraction, Feature Selection, and Radiomics Signature Construction

Our study followed and adhered to the Image Biomarker Standardization Initiative (IBSI) guidelines (30), and the software used was IBSI-compliant. The medical images were resampled to the 1 mm × 1 mm × 1 mm voxel size in millimeters before the subsequent feature extraction steps. The interpolator used for resampling was B-spline interpolation. For discretization of the image gray levels, the bin width was set as 25 for CT and 0.1 for PET-SUV. After preprocessing, a total of 1,672 × 2 radiomics features were extracted from the CT and PET images by the radiomics prototype after imaging preprocessing. The extracted radiomics feature groups were as follows: a) 18 first-order features, b) 16 size and shape features, and c) 74 texture encoding features. In total 1,672 radiomics features were extracted from each per lesion, including 108 from the original image, 460 [92 × 5] from the LoG-filtered images, 736 [92 × 8] from the wavelet-transformed images, and 368 [92 × 4] from non-linear intensity transforms (For detailed feature calculation formulas, please refer to the website: https://pyradiomics.readthedocs.io/en/latest/features.html#). A Spearman’s correlation test was performed using the ‘findCorrelation’ function in the caret package (cutoff, 0.9) to reduce feature redundancy. The least absolute shrinkage and selection operator (LASSO) Cox regression method, which is suitable for the regression of high dimensional data in survival analyses, was conducted to select the most useful predictive features from the training cohort (31). A radiomics score (rad score) was calculated for each patient *via* a linear combination of selected features that were weighted by their respective coefficients (32).

### Prognostic Model Establishment

The clinicopathological factors were analyzed using univariate Cox proportional hazards (CPH) regression analysis to identify significant risk factors. Significant risk factors with *p* < 0.05 were analyzed using the Kaplan–Meier curve and log-rank test. Significant risk factors were analyzed using multivariate Cox proportional hazards (CPH) regression analysis to identify independent risk factors. A clinical model was constructed based on the independent risk factors. Rad score and independent risk factors were fused into a single predictive model based on a multivariate CPH model. The performance of models was evaluated with the concordance index (C-index).

### Construction of the Radiomics Nomogram and Its Performance

The rad score and independent risk factors were based on multivariate Cox regression analysis to construct the radiomics nomogram. The prediction performance of the radiomics nomogram was assessed using the Harrell’s C-index in the training and validation cohorts. The C-index ranges from 0.5 to 1.0, where 0.5 indicates random data distribution and 1.0 suggests that the outcome of the model predicted the observed survival information perfectly. Calibration curves of the radiomics nomogram were then drawn for 5-year OS of the patients (33). The calibration curves illustrated both survival probabilities predicted by nomogram and the observed probabilities. A decision curve analysis determined the clinical usefulness of the radiomics nomogram by quantifying the net benefits at various threshold probabilities.

### To Guide the Individualized Targeted Therapy for Patients With Lung Adenocarcinoma

Patients with an *EGFR* mutation were divided into high- and low-risk groups according to their nomogram score. The treatment strategy was explored separately in the high- and low-risk cohorts using Kaplan–Meier analysis and a log-rank test, to find the cohort that would benefit from the targeted treatment. Additionally, the various treatment strategies were explored in patients with different EGFR-mutation sites, to identify which patients could actually benefit from adjuvant therapy.

### Statistical Analysis

The R software (version 3.5.0, www.Rproject.org) was used for all statistical analyses in this study. LASSO was conducted using the ‘glmnet’ package, while ‘hdnom’ was used for survival analysis. All statistical tests were two-sided and the significance level was set at *p* = 0.05.

## Results

### Clinical Characteristics

Patient characteristics of the training and validation cohorts were summarized in **Table 1**. There were no significant differences in age, sex, smoking status, lymph node metastasis, or distant metastasis, *etc.*, between the two cohorts (*p* > 0.05).

### Important Radiomics Feature Selection and Radiomics Signature Construction

**Figure 2:**
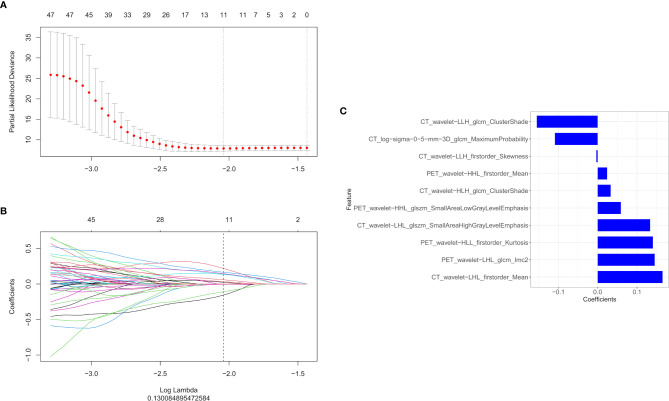
The LASSO and ten-fold cross-validation were used to extract the optimal subset of radiomics features. The following two steps were included: determining the hyperparameter/lambda with a partial likelihood deviance as the criterion **(A)** and using the optimized/lambda (the vertical dashed line) to select features with non-zero coefficients **(B)**. **(C)** LASSO algorithm was used to select the ten radiomics features that contributed the most to the prognostic prediction model.

In total, 1,672 radiomics features were extracted from the CT and PET images, respectively. We performed feature selection using the LASSO regression model with the PET/CT features (**Figures 2A, B**). The following ten important features were selected from 1,672 radiomics features (**Figure 2C**):

CT_wavelet−LLH_glcm_ClusterShade, CT_log−sigma−0−5−mm−3D_glcm_MaximumProbability,
CT_wavelet−LLH_firstorder_Skewness,PET_wavelet−HHL_firstorder_Mean,CT_wavelet−HLH_glcm_ClusterShade,PET_wavelet−HHL_glszm_SmallAreaLowGrayLevelEmphasis,CT_wavelet−LHL_glszm_SmallAreaHighGrayLevelEmphasis,PET_wavelet−HLL_firstorder_Kurtosis,PET_wavelet−LHL_glcm_Imc2, and CT_wavelet−LHL_firstorder_Mean.
Then the rad score was calculated using these ten radiomics features as follows: rad score = 0.051*PET_wavelet-HLL_firstorder_Kurtosis+0.006*PET_wavelet-HLL_glcm_Idn+-0.011*PET_wavelet-LHH_glcm_Imc1+0.047*PET_wavelet-LHL_glcm_Imc2+-0.011*PET_log-sigma-0-5-mm-3D_glszm_SmallAreaLowGrayLevelEmphasis + 0.093.

### Prognostic Model Establishment and Performance of the Multimodality Prediction Model

**Figure 3:**
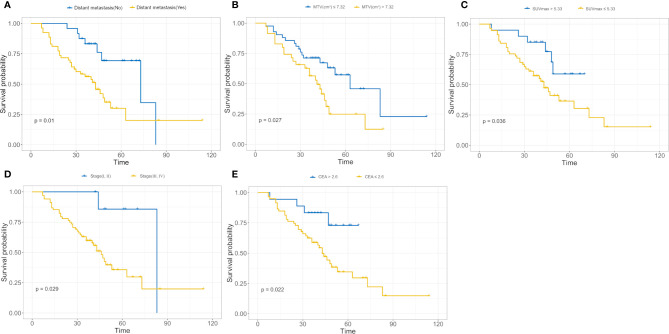
**(A–E)** Kaplan**–**Meier analysis for distant metastasis **(A)**, metabolic tumor volume (MTV) **(B)**, maximal standard uptake value (SUVma_x_); **(C)**, stage **(D)**, carcinoembryonic antigen (CEA); **(E)**. The patients were stratified into high- and low-risk groups based on distant metastasis (**A**, *p* = 0.01, log-rank test), MTV (**B**, *p* = 0.027, log-rank test), SUVmax (**C**, *p* = 0.036, log-rank test), stage (**D**, *p* = 0.029, log-rank test), and CEA (**E**, *p* = 0.022, log-rank test).

We used a univariate Cox regression analysis to test the hazard ratio (HR) of each factor and to determine its’ significance in the probability of death. The results were as follows: distant metastasis (HR, 2.68), metabolic tumor volume (MTV, HR, 2.02), maximal standard uptake value (SUVmax, HR, 2.48), stage (HR, 4.29), and carcinoembryonic antigen (CEA, HR, 3.16) were the significant risk factors for patients with an *EGFR* mutation (*P* < 0.05).The significant risk factors with *p* < 0.05 were calculated using a log-rank test, and Kaplan–Meier curves were plotted. **Figures 3A–E** illustrate the survival probability of patients in the high-risk or low-risk cohorts. The results of the log-rank test indicate significant discrimination between the two groups. A clinical model was constructed based on multivariate Cox proportional hazards (CPH) regression analysis of significant risk factors. Distant metastasis [HR,2.97(95%CI, 1.36–6.51)] and metabolic tumor volume [MTV,HR,2.26(95%CI, 1.19–4.28)] were the independent risk factors in the training cohort. Rad score and independent risk factors were fused into a single predictive model based on the multivariate CPH regression analysis. Distant metastasis [HR,1.80(95%CI, 0.80–4.04)], metabolic tumor volume [MTV, HR,1.62(95%CI, 0.82–3.17)] and rad score [HR,17.23 (95%CI, 6.62–44.81)] were the independent risk factors in the training cohort. The C-index of the clinical model was 0.694 and 0.729 in the training and validation cohorts, respectively. The C-index of the rad score (radiomics model) was 0.819 and 0.737 in the training and validation cohorts, respectively. A rad score was combined with the independent risk factors to construct a combined model (radiomics nomogram) based on multivariate Cox regression analysis, and the C-index of the combined model (radiomics nomogram) was 0.840 and 0.803 in the training and validation cohorts, respectively (**Table 2**).

**Table 2:** The comparison of prognostic accuracy between the radiomics model and two other prognostic models.

models	Training cohort		Validation cohort
	C-index 95% CI		C-index 95% CI
Radiomics model	0.819(0.764–0.874)		0.737(0.606–0.868)
Clinical model	0.694(0.618–0.770)		0.729(0.599–0.858)
Radiomics nomogram	0.840(0.787–0.893)		0.803(0.689–0.917)

### Development of the Radiomics Nomogram and Its Performance

**Figure 4:**
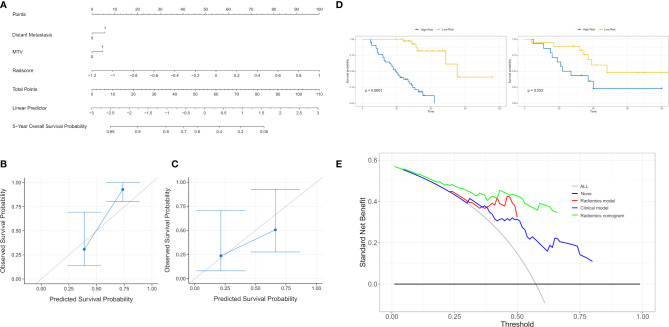
**(A)** A radiomics nomogram for prediction of 5-year overall survival for patients with lung adenocarcinoma of EGFR mutations. **(B)** Calibration curve of the radiomics nomogram in the training cohort. **(C)** Calibration curve of the radiomics nomogram in the validation cohort. Calibration curve for the estimation of 5-year overall survival as predicted by the nomogram. The nomogram-estimated overall survival is plotted on the x-axis, and the actual overall survival is plotted on the y-axis. Dash line represents an ideal agreement. **(D)** The Kaplan–Meier curve showed that this nomogram score could effectively discriminate high-risk patients from low-risk patients. **(E)** The decision curve analysis for each model. The y-axis denotes the net benefit, which was calculated using true-positive and false-positive results. The radiomics nomogram model has the highest net benefit at the threshold from 0.1 to 0.9 among all positive predictions (line labeled “All”); all negative predictions (line labeled “None”) and two other clinical models (line labeled “Radiomics model and clinical model”).

The rad score was combined with the independent risk factors to construct a radiomics nomogram based on multivariate Cox regression analysis (**Figure 4A**). The C-index of the radiomics nomogram was 0.840 and 0.803 in the training and validation cohorts, respectively. The calibration curve result showed that the predicted probability was remarkably close to the actual survival time of patients (**Figures 4B, C**). Kaplan–Meier survival analysis of patients in the high-risk and low-risk groups in the training cohort (log-rank test *p* = 0.001; **Figure 4D**). A decision curve analysis showed that the radiomics nomogram had a higher overall net benefit than the clinical model and the radiomics model, and had a higher overall net benefit across the majority of the range of reasonable threshold probabilities (**Figure 4E**).

### To Guide the Targeted Treatment for Lung Adenocarcinoma in Patients With EGFR Mutations

**Figure 5:**
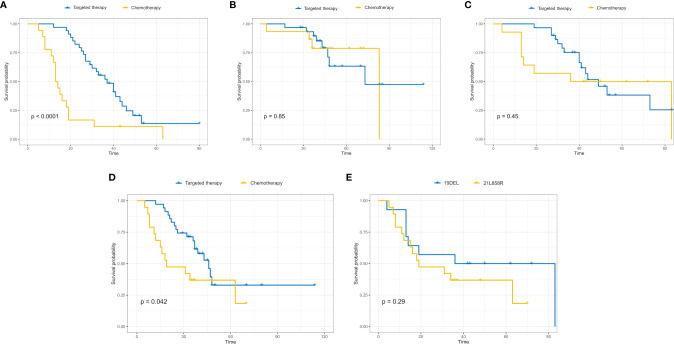
High-risk patients with *EGFR* mutations had a higher sensitivity to targeted therapy (*p* < 0.0001) **(A)**, while the sensitivity of low-risk patients to targeted therapy and chemotherapy was not significantly different (*p* = 0.85) **(B)**. In high-risk patients, the sensitivity of patients with the 19DEL mutation to chemotherapy and targeted therapy was not significantly different (*p* = 0.45) **(C)**, while the sensitivity of patients with the 21L858R-mutation to chemotherapy and targeted therapy was significantly different (*p* = 0.042) **(D)**. **(E)** The sensitivity of patients with the 19DEL- and 21L858R-mutations to chemotherapy was not significantly different (*p* = 0.29).

According to the cut-off value of nomogram score at 0.369, the corresponding 5-year overall survival probability was 0.58. Patients with an *EGFR*-mutation were divided into high- and low-risk groups, and the sensitivity of high- and low-risk patients to chemotherapy and targeted therapy was analyzed. The results showed that high-risk patients had a higher sensitivity to targeted therapy (*p* < 0.0001), indicating that targeted therapy is the main treatment method for patients with high-risk *EGFR* mutations, while the prognoses of the two therapies were similar in the low-risk group (*p* = 0.85, **Figures 5A, B**). In patients with an 19DEL mutation, there was no significant difference in the sensitivity to chemotherapy and targeted therapy (*p* = 0.45). The patients with a 21L858R-mutation had significant differences in sensitivity to chemotherapy and targeted therapy, and the patients with a 21L858R-mutation were more likely to benefit from targeted therapy (*p* = 0.042; **Figures 5C, D**). In addition, there was no significant difference between patients with a 19DEL-mutation and patients with a 21L858R-mutation in their benefit from chemotherapy (*p =* 0.29; **Figure 5E**).

## Discussion

In our study, we developed a radiomics nomogram based on ^18^F-FDG PET/CT radiomics features combined with clinicopathological factors to predict survival outcomes in patients with lung adenocarcinoma of EGFR mutations, with the aim of providing guidance for personalized targeted treatment of patients with lung adenocarcinoma with EGFR mutations.

In the CPH model for evaluating the prognosis of patients with *EGFR* mutations, distant metastasis, MTV, stage, CEA, and SUVmax were the significant prognostic risk factors. Among them, the patient’s risk of death was higher when the patient had MTV (>7.32). MTV is a parameter that reflected the metabolic burden of the whole-body tumor compared with other PET/CT semiquantitative parameters and related clinicopathological factors. It can more effectively stratify the risk of patients and identify high-risk groups. In particular, it can effectively evaluate the prognosis of patients with advanced lung cancer. This was consistent with our findings (34, 35). SUVmax is the most used metabolic parameter of PET/CT in clinical work and only represents a single pixel value of the tumor metabolism that is most active in the outlined area. Whether SUVmax is an independent risk factor for lung cancer remains controversial (22). Some studies believe that SUVmax can effectively indicate the degree of tumor differentiation and provide evidence for the prognosis of patients (36). Our study demonstrated that when SUVmax (>5.33), the patient’s risk of death increased. This was consistent with our findings.

In addition, we combined the rad score with independent risk factors (Distant metastasis and MTV) based on multivariate Cox regression analysis to construct a radiomics nomogram that predicted survival outcomes of patients with EGFR mutations. The results showed that a radiomics nomogram can predict survival outcomes very well. Its’ C-index was 0.840 and 0.803 in the training and validation cohorts, respectively, which could stratify high- and low-risk groups quite well. At present, few studies based on PET/CT radiomics have predicted the survival of lung cancer patients with *EGFR* mutations, and their predictive performances were generally poor (37, 38). Kirienko et al. (28) used radiomics signatures based on PET/CT to predict disease-free survival (DFS) of patients with NSCLC after surgery. The results showed that the AUC of the Cox model based on the radiomics signature was 0.68, and the AUC was 0.65 after combining it with clinical predictors. Moreover, the current study focused mainly on a CT modality while predicting survival, and the value of the C-index was usually not well *i.e*., did not exceed 0.70. The performance improved after combining it with clinicopathological factors (39, 40). Our results showed that the C-index reached 0.803, and our result was a small breakthrough in the results of previous studies. To guide the treatment of patients with *EGFR* mutations, our study analyzed the effects of different treatment strategies on the prognosis of patients with *EGFR* mutations. Our results showed that the rad score could stratify patients with *EGFR* mutations into high- and low-risk groups. For patients who were at high risk, targeted therapy is recommended to improve survival. For patients at low risk, there was no significant difference in survival regardless of whether targeted therapy or chemotherapy was chosen. The patients with a 21L858R-mutation had significant differences in sensitivity to chemotherapy and targeted therapy, and the patients with a 21L858R-mutation were more likely to benefit from targeted therapy. However, in patients with a 19DEL mutation, there was no significant difference in the sensitivity to chemotherapy and targeted therapy. It may be due to the small sample size and the bias caused by retrospective study. In addition, there was no significant difference between patients with a 19DEL mutation and patients with a 21L858R-mutation in their benefit from chemotherapy. It illustrated that patients with EGFR mutations may not benefit from chemotherapy. Our results indicated that radiomics features could identify patients who are more likely to benefit from targeted therapy among patients with EGFR mutations, and would benefit from treatment guidance.

Our study had many strengths. First, our study not only predicted survival outcomes in lung adenocarcinoma patients with *EGFR* mutations, but also identified patients with EGFR mutations who were likely to benefit from targeted therapy through rad score. We provided guidance for the selection of treatment methods in patients with *EGFR* mutations, which was rarely reported in previous studies. Second, patients in this study were scanned using the same PET/CT device used in a standard protocol, which avoided the heterogeneity of image impressions caused by the use of different scans and reconstruction parameters. This led to more stable and reliable results.

Our study had some limitations. First, this was a retrospective study with a small data set and no external validation, which may have introduced selection bias. Second, we only studied the effect of treatment on the prognosis of patients with lung adenocarcinoma and an *EGFR* mutation status and did not consider the influence of other genes. Further studies are essential to evaluate other genes comprehensively.

In conclusion, a ^18^F-FDG PET/CT rad score combined with clinicopathological factors can predict the survival outcomes of patients with lung adenocarcinoma with an *EGFR* mutation. This novel and non-invasive approach can be provide with a more precise imaging diagnosis and personalized treatment guidance for patients with an EGFR mutant and have a significant clinical application value.

## Data Availability Statement

The raw data supporting the conclusions of this article will be made available by the authors, without undue reservation.

## Ethics Statement

The institutional review board of Affiliated Jinling Hospital, Medical School of Nanjing University approved this retrospective study and waived the need to obtain informed consent from the patients.

## Author contributions

BY conceived the idea of the study. BY, HJ, JinZ, LM, JiaZ, HD, and CZ collected the data. HZ and GL performed image analysis. BY wrote the manuscript. SD performed the statistical analysis. CZ, JT, LZ, FW, and GL edited and reviewed the manuscript. All authors contributed to the article and approved the submitted version.

## Funding

This work was supported by the National Key Research and Development Program of China (2017YFC0113400 for LZ) and Natural Science Foundation of Jiangsu Province (BK2011665).

## Conflict of Interest

S-fD was employed by GE Healthcare China.

The remaining authors declare that the research was conducted in the absence of any commercial or financial relationships that could be construed as a potential conflict of interest.

## Abbreviations

CEA, carcinoembryonic antigen; EGFR, epidermal growth factor receptor; MTV, metabolic tumor volume; NSCLC, non-small cell lung cancer; PET/CT, positron emission tomography/computed tomography; SUVmax, maximal standard uptake value; SUV_mean_, mean standard uptake value; TKIs, tyrosine kinase inhibitors; TLG, total lesion glycolysis.
