# Supplementary material for: Value of 18F-FDG PET/CT-Based Radiomics Nomogram to Predict Survival Outcomes and Guide Personalized Targeted Therapy in Lung Adenocarcinoma With EGFR Mutations
Source: Front Oncol. 2020 Nov 11;10:567160. doi: 10.3389/fonc.2020.567160 (PMC7686546; doi:10.3389/fonc.2020.567160)
Supplement: Supplementary file 1 [file DataSheet_1.pdf]

Tumor segmentation

A volume of interest(VOI)was drawn semiautomatically around the tumor by a chest radiologist (Y.B., 9 years of experience) in the lung diagnosis using the radiomics prototype (Radiomics, Frontier, Siemens) and confirmed by another chest radiologist (J.H.S., 15 years of experience). Both radiologists were blinded to the patients' clinical information.Firstly,we import CT images into radiomics prototype software (Radiomics, Frontier, Siemens).In the segmentation module, a few segmentation tools were available for semiautomatic delineation of the tumor in three dimensions. The segmentation was semiautomatically produced by drawing a line across the boundary of the tumor, then, the tool automatically find the neighboring voxels in 3D space with the same gray level through an automatic algorithm, and this is a Random Walker-based lesion segmentation for solid and subsolid lung lesions. The first step is to obtain a superset of voxels that may be part of the lesion.This can be implemented efficiently as a 3 D region growing starting from the center of the ROI. Then the thresholds can be fixed for lesions or determined adaptively from an analysis of the density distribution in the ROI. The region growing results in complete lesion and additionally parts of the attached vasculature. A morphological opening operation is applied to remove the vessels finally. If the segmentation wasn't right, the operators could correct it manually in the 3D domain using the radiomics prototype. The algorithm aimed at K-way image segmentation with given seeds indicating regions of the image belonging to the K objects(the objects to be segmented). Each seed specifies a location with a user-defined label. The algorithm labels an unseeded pixel by resolving the question: Given a random walker starting at this location, what is the probability that it first reaches each of the K seed points? It will be shown that this calculation may be performed exactly without the simulation of a random walk. By performing this calculation, we assign a K-tuple vector to each pixel that specifies the probability that a random walker starting from each un- seeded pixel will first reach each of the K seed points. A final segmentation may be derived from these K-tuples by selecting for each pixel the most probable seed destination for a random walker. By biasing the random walker to avoid crossing sharp intensity gradients, a quality segmentation is obtained that respects object boundaries (including weak boundaries). And then in the radiomics module to click the computer features tool to calculate the CT radiomic features, and export the CT Masks+STL at the same time. Then we import the PET image into the software. If the tumor on the PET image is not at the same slice as the CT, we manually adjust the slice of PET image. Then, the CT Masks+STL will be imported into the software to cover the tumor on the PET image. If the CT Masks+STL does not cover the tumor, two radiologists(Y.B; J.HS) manually adjusted the CT Masks+STL through edit tools and reached a consensus to ensure that the CT Masks+STL completely covered the tumor lesions on the PET image as much as possible, and then use the same method to extract PET radiomic features.So, the 3D ROI (VOI) was delineated on CT image, and could be used by the PET image when the PET image were transformed to the CT image space using the transformation matrix obtained in PET-CT fusion.
